# Supplementary material for: Programme of triple-I mediator education (TIME) to improve medical disputes in clinical settings in Taiwan: a Delphi study
Source: BMJ Open. 2022 Aug 26;12(8):e058880. doi: 10.1136/bmjopen-2021-058880 (PMC9422892; doi:10.1136/bmjopen-2021-058880)
Supplement: Author's manuscript [file bmjopen-2021-058880.draft_revisions.pdf]

BMJ Open

BMJ Open is committed to open peer review. As part of this commitment we make the peer review history of every article we publish publicly available.

When an article is published we post the peer reviewers' comments and the authors' responses online. We also post the versions of the paper that were used during peer review. These are the versions that the peer review comments apply to.

The versions of the paper that follow are the versions that were submitted during the peer review process. They are not the versions of record or the final published versions. They should not be cited or distributed as the published version of this manuscript.

BMJ Open is an open access journal and the full, final, typeset and author-corrected version of record of the manuscript is available on our site with no access controls, subscription charges or pay-per-view fees (http://bmjopen.bmj.com).

If you have any questions on BMJ Open's open peer review process please email info.bmjopen@bmj.com

BMJ Open

From cacophony to euphony: Time for Medical Dispute

Journal:	*BMJ Open*
Manuscript ID	bmjopen-2021-058880
Article Type:	Original research
Date Submitted by the Author:	03-Nov-2021

Complete List of Authors:	Shiao, Yi-Chih; Tri-Service General Hospital, Department of Family and Community Medicine; National Defense Medical Center<br>Shen, Ruo-Nan; Taiwan Drug Relief Foundation<br>Chen, Wen-Wen; Taiwan Drug Relief Foundation<br>Liu, Yeh-Ping; Department of Medical Affairs; Taiwan Ministry of Health and Welfare<br>SHIH, Chung-Liang ; Taiwan Ministry of Health and Welfare<br>Wang, Chih-Chia; Tri-Service General Hospital, Department of Family and Community Medicine; National Defense Medical Center

Keywords:	Health policy < HEALTH SERVICES ADMINISTRATION & MANAGEMENT, MEDICAL EDUCATION & TRAINING, PUBLIC HEALTH

SCHOLARONE™
Manuscripts

I, the Submitting Author has the right to grant and does grant on behalf of all authors of the Work (as defined in the below author licence), an exclusive licence and/or a non-exclusive licence for contributions from authors who are: i) UK Crown employees; ii) where BMJ has agreed a CC-BY licence shall apply, and/or iii) in accordance with the terms applicable for US Federal Government officers or employees acting as part of their official duties; on a worldwide, perpetual, irrevocable, royalty-free basis to BMJ Publishing Group Ltd ("BMJ") its licensees and where the relevant Journal is co-owned by BMJ to the co-owners of the Journal, to publish the Work in this journal and any other BMJ products and to exploit all rights, as set out in our licence.

The Submitting Author accepts and understands that any supply made under these terms is made by BMJ to the Submitting Author unless you are acting as an employee on behalf of your employer or a postgraduate student of an affiliated institution which is paying any applicable article publishing charge ("APC") for Open Access articles. Where the Submitting Author wishes to make the Work available on an Open Access basis (and intends to pay the relevant APC), the terms of reuse of such Open Access shall be governed by a Creative Commons licence – details of these licences and which Creative Commons licence will apply to this Work are set out in our licence referred to above.

Other than as permitted in any relevant BMJ Author's Self Archiving Policies, I confirm this Work has not been accepted for publication elsewhere, is not being considered for publication elsewhere and does not duplicate material already published. I confirm all authors consent to publication of this Work and authorise the granting of this licence.

For peer review only

From cacophony to euphony: Time for Medical Dispute

Yi-Chih Shiao[1,2], Ruo-Nan Shen[3], Wen-Wen Chen[3], Yueh-Ping Liu[4,5], Chung-Liang Shih[5], Chih-Chia Wang[1,2*]

*Correspondence: Chih-Chia Wang

Address: Number 325, Section 2, Chenggong Rd, Neihu District, 114, Taipei, Taiwan.

E-mail: tsghccwang@gmail.com

Tel: +886-2-87923311 ext. 16567

Fax: +886-2-87927057

[1]Division of Family Medicine, Department of Family and Community Medicine, Tri-Service General Hospital; No. 325, Section 2, Chenggong Rd, Neihu District, Taipei City 114, Taiwan (R.O.C.)

[2]School of Medicine, National Defense Medical Center, Taipei, Taiwan (R.O.C.); No. 161, Section 6, Minquan E Rd, Neihu District, Taipei City 114, Taiwan (R.O.C.)

[3]Taiwan Drug Relief Foundation, Taipei, Taiwan (R.O.C.); 10F., No. 22, Aiguo E. Rd., Zhongzheng Dist., Taipei City 100, Taiwan (R.O.C.)

[4]Department of Medical Affairs, Ministry of Health and Welfare, Taipei, Taiwan (R.O.C.); No. 488, Section 6, Zhongxiao E Rd, Nangang District, Taipei City 115, Taiwan (R.O.C.)

[5]Ministry of Health and Welfare, Taipei, Taiwan (R.O.C.); No. 488, Section 6, Zhongxiao E Rd, Nangang District, Taipei City 115, Taiwan (R.O.C.)

Keywords: Alternative dispute resolution, Delphi method, in-house mediation, training programme syllabus

For peer review only

From cacophony to euphony: Time for Medical Dispute

Manuscript word count: 2984 words

Abstract word count: 191 words

For peer review only

From cacophony to euphony: Time for Medical Dispute

Abstract

Objectives To establish a training programme to cultivate trainee mediation skills through time investment, skill incorporation, and formation of in-house mediation services.

Design A four-day consensus conference was conducted to determine core competences and relevant curricula through the four-round modified Delphi process.

Setting Responses collected through four rounds of the Delphi process from 11th November 2018 to 17th May 2019.

Participants Onboard mediators with different specialties.

Outcome measures Items with a median rating of 4 or more on a Likert scale of 1 to 7 points and 70% or more in agreement were identified as core competence and curricula.

Results The training syllabus was set up based on agreement from round 1 to 4 and included four pillars of core competence, such as 'knowledge base of law', 'internalisation of the denotative and connotative meanings of care', 'effective, smooth, and timely communication', 'conflict resolution', and corresponding training curricula.

Conclusions The consensus conference developed a syllabus to train apprentices to manage medical disputes and improve the relationship between patients and medical staff. Policymakers in healthcare and top management in healthcare institutions can use this syllabus to guide their future education and training programme.

For peer review only

Strengths and limitations of this study

➢ The panel group comprised highly experienced experts with different specialities in mediation. This composition facilitated the comprehensive establishment of a teaching structure.

➢ The syllabus could guide the naive trainers to raise a training programme systemically under a fully constructed theoretical framework.

➢ A retrospective follow-up to evaluate the effectiveness of training outcome was lacking and a periodic assessment would be favourable.

For peer review only

Introduction

The number of medical disputes has been increasing over the past two decades[1]. Post-dispute problem-solving strategies included in-court conventional litigation and out-of-court alternative dispute resolution (ADR)[2-4]. ADR has been integrated into the healthcare system of many countries[5-7] for mitigating risk of being sued and making both patients and physicians satisfied[2, 3]. Pre-dispute risk identification and early intervention, however, was merely merged into our current system.

In Taiwan, the triple-I mediator (TIME) task force was designed to address dispute prevention. TIME is a multi-disciplinary team with voluntary mediators in several professions at different levels of healthcare institutions and in different geographic regions. Policymakers draw on the concept of medical emergency team[8, 9] or rapid response team[10] for the purpose of high acuity response to early deterioration of relationships between patients and physicians. TIME task force is characterized by "in-house argument handling", "immediate action", "integration resources", and is initiated by the Taiwan Ministry of Health and Welfare (MOHW) on the legal basis of Article 82 of the Medical Care Art.

The onboard mediators in TIME task forces are seasoned experts and have been seed teachers in their separate hospitals. However, there is no systematic course for personnel training in the long run. To maintain good medical quality and develop a sustainable operation on appeasing conflicts, Taiwan launched a five-year TIME Quality Improvement Programme in 2018. The purpose of this article is to present the syllabus for the course of TIME training through the Delphi method.

For peer review only - http://bmjopen.bmj.com/site/about/guidelines.xhtml

Methods

Patient and public involvement

No patients and public were not involved in this study.

The Delphi method

The Delphi method is reliable and has been effectively used for establishing consensus regarding healthcare management.[11] It is a multistage, iterative, and anonymous process consisting of at least two rounds of surveys.[12] Considering the critical paucity of relevant evidence for fostering skills, the Delphi method with four rounds of surveys was introduced to achieve consensus regarding the syllabus for the TIME programme. After a review of research studies and preliminary agreement between panel members, initial rounds of open-ended questions elicited various thoughts and perspectives. In the following rounds, the entire panel provided a summary of feedback and asked members to revise their responses. The strengths of this method include anonymity, capability of obtaining a collection of ideas and opinions from participants, and cost effectiveness. The weaknesses were the length of time required, research bias through the potential influence of moderators or participants, and uneven distribution of specialties among the respondents. The authors followed the Standards for Quality Improvement Reporting Excellence (SQUIRE 2.0) guidelines.[13]

Moderator and panel members

Delbecq *et al* suggested that 10–15 members could be a satisfactory range for a panel group.[14] Our goal was to have at least 10 participants for each round. The list of professionals of interest, approved by Taiwan's MOHW, was followed in panel recruitment through direct invitation by the Taiwan Drug Relief Foundation (TDRF), a government-organised nongovernmental organisation. The moderators were general practitioners with Doctor of Law degree who were devoted to the integration of medical and law fields in the TDRF through collaboration with a lawyer with expertise in medical law. The panel comprised six

For peer review only

doctors from different medical specialties (e.g., gastroenterology, obstetrics and gynaecology, dermatology, emergency medicine, otolaryngology, and genitourinary medicine) at different levels of healthcare facilities, two lawyers, one judge, one clinical psychologist, and one social worker. All members had >10 years of experience in mediation.

Agreement and consensus

Agreement was defined as the percentage of positive rather than neutral or negative responses divided by the total completed responses. Consensus was defined as 70% or more agreement according to the recommendations made by Thelma Sumsion.[15]

Delphi survey rounds

The Delphi survey comprised four rounds with consistent anonymity in each round. Opinions and feedback were structurally categorised under different headings after each round and exported to a temporary meeting document for reference to effectively facilitate discussions among moderators in the next round. The 4-day, in-person, Consensus Conference was conducted on November 11, 2018, January 1, March 11, and May 17, 2019, separately, to identify and achieve consensus on research gaps.

In round 1, an open-ended questionnaire was initiated, and respondents were requested to provide their job description, relevant experience, and professional knowledge regarding identifying and prioritising issues before analysing a problem, devising a strategy, and implementing plans in the given period during dispute resolution and retrospectively evaluating communication effectiveness through self-reflection. The group considered whether they exhibited pillars of professionalism in the TIME programme based on relevant content in medical education,[16] Roach's Six C's in caring,[17] and a communication model called CI-CARE proposed by the University of California, Los Angeles Health System,[18] as a benchmark. Opinions and details on constructing core competences correlated with fostering

For peer review only

professionalism were provided using narrative text responses and discussion. The aforementioned information was carefully gathered to generate a draft questionnaire.

In round 2, the draft proposal comprising several features of core competences was presented and measured through a questionnaire answered using a 7-point Likert-type scale (1 = *strongly disagree*, 2 = *disagree*, 3 = *somewhat disagree*, 4 = *neutral*, 5 = *somewhat* agree, 6 = *agree*, and 7 = *strongly* agree). Consensus was based on an agreement level of >70%. Items with <70% agreement and relevant feedback regarding disagreement with a core competence was discussed by the moderator group. Participants were then requested to share opinions and details for constructing the core curricula correlating to fostering core competences by using narrative text responses. Information on positive and negative aspects mentioned earlier were gathered systematically to generate the initial draft containing the core competences and core curricula of the TIME programme.

In round 3, agreement among the panel members regarding the draft of the core curricula was obtained using a 7-point Likert scale similar to that used in round 2 with respect to the level of agreement by the respondents. Consensus was reached and discussions were held by the moderator group if any item had <70% agreement. Additional narrative text was provided to determine whether any disagreement existed regarding the core curriculum. This draft was revised by the group based on the analysis of agreement and annotation of round 3.

In round 4, respondents were asked to provide suggestions on syntactic errors, improper phrasing, or terminology to make the description concise. The final version of the syllabus, called triple-I health mediator (TIME) training programme including (1) immediate action; (2) policy incorporation; (3) in-house settlement, was based on agreement in rounds 2 and 3; explanations for any revision were presented by the working group and announced on 15 November 2019, which was the promulgation date (Figure 1).

For peer review only - http://bmjopen.bmj.com/site/about/guidelines.xhtml

Results

The characteristics of the participants are presented in Table 1. All the 11 participants completed the four-round Delphi survey. The expert panel group consisted of 7 men (63.6%) and 4 women (36.4%). Physicians predominated the expert panel group (54.5 %). The respondents enrolled were experienced, knowledgeable, and skilful specialists of different fields possessing more than 10 -year experience.

Table 2 shows a consensus on all items, with more than 80% agreement. The item 'effective, smooth, and timely communication' had the highest number of responses of 'strongly agree' (n = 10, 90.9%), and the rest of the items, including 'knowledge base of law', 'internalisation of the denotative and connotative meanings of care', and 'conflict resolution', had almost equal agreement (n = 6, 54.5%). Only the item 'knowledge base of the law' elicited a neutral opinion (n = 2, 18.2%).

In table 3, a consensus was reached for all items, with an agreement of 100%, except for the item 'contentious proceeding in law case with medical errors: conventional means', which had 91.7% agreement. Only in the 'knowledge base of law' category was the number of participants who responded of strongly agree below 10 (n = 9). The other three categories had at least one item with >10 people indicating strong agreement. Additionally, in the categories of 'effective, smooth, and timely communication' and 'conflict resolution', consensus was reached on all of the correlated core curricula, with more than half of the members in strong agreement. Furthermore, only the category of 'effective, smooth, and timely communication' had two items for which 10 members were in strong agreement, namely 'ethics of TIME: knowing the difference between what you have a right to do and what is right to do' and 'Establishment of communication: an impregnable foundation leaving no boundary and obstacle.

For peer review only

Discussion

After a four-round Delphi process was applied, consensus was reached regarding the syllabus for the TIME training programme, which was established by a panel consisting of highly experienced experts in mediation. Table 4 was the final product containing four items on core competencies with respect to relevant core curricula. Each item received at least 80% agreement, which represents convergence of opinions.

Increases in medical disputes and civil and criminal proceedings: the current situation in Taiwan

According to the latest statistical data from Taiwan, the number of conflicts increased 3-fold from 145 in 1987 to 474 in 2015.[19] Moreover, controversial cases could be subcategorised into civil and criminal ones. Both civil and criminal cases increased at a tremendous speed from 128 and 4 in 1987 to 355 and 115 in 2015, respectively.[20] After factual approach of causation in retrospective analysis, the results were beyond our expectations that non-negligence related dispute was majority (e.g. 112 out of 145 in 1987 and 403 out of 474 in 2015) rather than negligence-induced ones all the time.[21] The aforementioned information showed increased trends related to conflicts indicated a need to discover coping strategies for growing numbers of disputes.[22]

In-house mediation (IHM): corresponding strategies for dispute resolution

To optimise the present healthcare system, Taiwan's MOHW introduced an IHM system for conflict management within the organisation in 2013.[23] According to current regulations on IHM in Taiwan, mediators are defined as sentinel, caring people who provide mediation for a suspected or established dispute and collaborate with team members from different specialties depending on the scale or type of dispute. Of the total number of members in the team, at least three must be in-house medical staff in addition to the original teams of healthcare providers and nonmedical staff (e.g., legal professionals or social workers).

For peer review only - http://bmjopen.bmj.com/site/about/guidelines.xhtml

Nonmedical staff should comprise at least one third of the team. The design of IHM was aimed at creating a 360° feedback mechanism and obtaining good understanding of excellence; in reality, the ultimate goal of IHM application in dispute resolution was to ensure highly effective teamwork for appropriate dispute resolution rather than blame shifting.

Timely presence of an empathetic person: the most valuable intervention

A mediator, considered valuable in an interdisciplinary team, can play a role in diverse situations. In a community, they were recruited for motivating the participation of minority groups at health status assessment and facilitating entrance of primary care services.[24] In hospital settings, a mediator can engage with both parties of a dispute, integrate resources from, for example, Taiwan's drug relief foundation, and offer a timely intervention to patients if they encounter difficulty or discomfort or are involved in a dispute related to medical practice. The core premise of this intervention is effective communication with perfect reciprocal understanding. However, incredulity arises from poor communication rather than incompetency,[25] and it occurs because of a breakdown in the doctor–patient relationship, leading to defensive medicine practice built as a nonviolent strategy in conflict.[26] Many factors contribute to communication improvement. For example, assistance with using online translation tools, such as Google Translate, could help overcome language barriers and improve satisfaction levels among healthcare providers and patients.[27] To completely mitigate tension and subsequent conflict, 'internalisation of the denotative and connotative meanings of care' is the core competence, and it is characterised as person-centred caring (PCC) in our curricula,[28] being a key factor in the delivery of high-quality healthcare.[29]

Barrier-free channels of communication

For peer review only - http://bmjopen.bmj.com/site/about/guidelines.xhtml

We expressed a strong desire to establish this platform to provide immediate emotional support and 'Effective, smooth, and timely communication' set the foundation for it. A consensus regarding the need of incorporation of mediation administration in cases as an inherent element of the training programme.[30] The ethics of TIME training encouraged inexperienced staff to exhibit empathy, keep an open mind, and be proficient in providing emotional support through situational exercise. Furthermore, the 'psychological analysis of patients' component helps inexperienced staff integrate diverse evidence in clinical settings and apply such evidence during each visit. Human emotions are too complex to describe through facial expressions, especially emotions such as grief or fear.[31] Describing someone's emotional journey through stages of the Kübler-Ross Change Curve is inappropriate.[32] However, emotional clues conveying messages about physical or mental health should be detected by a well-educated mediator to precisely and rapidly respond to such emptions because physical discomfort leads to mental and emotional suffering.[33] Different beliefs about religion[34] and education[35] lead to diverse reactions to health problems. With the assistance of mediators, each reaction can be directed to an appropriate and corresponding resolution.

Rebuilding confidence and trust in patients

Rebuilding patient's trust rationally and emotionally is an arduous task. For patients, physical health decline, either temporarily or permanently, often has mutual[36] and detrimental effects on psychological health (e.g., anxiety[37], depression[38]). To restore confidence and trust, the so-called 'transformative mediation'[39] with two key features, is fundamental. One is empowerment shift[40], which could allow both parties to regain respect, confidence, and empathy. The other is recognition shift[40], which could help patients reduce their defensiveness against medical staff and gain a different perspective. This approach made patients express needs and strengthened bilateral participation in health care issue.[41]

For peer review only - http://bmjopen.bmj.com/site/about/guidelines.xhtml

Looking after medical staff

Various needs of both physicians and patients necessitate the involvement of a third party. The potential emotional trauma of staff in response to adverse patient responses can cause burnout.[42] Such burnout has been thought high among physicians globally.[43] Consequences of burnout have detrimental effects on doctors, which may lead to them making incorrect decisions (thus causing medical errors), exhibiting a hostile attitude towards patients, and having dysfunctional relationships with colleagues.[42] Other environmental factors (e.g., mainstream media and politics) may lead to staff having traumatic experiences, resulting in second victim syndrome (SVS).[44] SVS cannot be treated if a systematic and well-structured support for coping with emotional burdens is absent.[45] In short, medical staff must exhibit a caring attitude.[46] Mediators could provide pressure relief and help conflicting parties overcome difficulties.

Utilizing resources effectively

Mediators make effective use of resources to provide timely relief to patients. In clinical settings, patients focus on discomfort and ignore or miss relevant information regarding available treatments. The utility of this related mediation may be time sensitive. Mediators accelerate the use of individualised services targeting specific demands after the overall evaluation of case needs and concerns. For those with legal liability for any damage they cause, legal remedies affirm the validity of their claims and requests. Damage caused by those with no liability could be dealt with through compensation.[47] Common access points to humanitarian relief include Taiwan's drug injury relief system, compensation for vaccine injuries, and childbirth accident relief.

Towards improving the current situation

Upgrading communication effectiveness in the current medical environment is inevitable for the growing numbers of dispute in the decades regardless of occupations. Effective

For peer review only - http://bmjopen.bmj.com/site/about/guidelines.xhtml

physician–patient communication serves to assist physicians in making precise diagnoses,[29] enhance patient satisfaction,[29] facilitate information exchange,[29] encourage patient participation,[29] and control diseases (physiologically or subjectively).[48] Communication in nursing practice is important to bridge knowledge gaps, helping nurses gain communication skills and a positive attitude.[49] Mediators could provide medical staff with guidance regarding well-tailored PCC services during the processes of settling disputes and conducting a retrospective assessment and these efforts may build a better environment.

Continuity of TIME: as TIME goes by

Persons with the following traits are considered qualified and eligible to be mediators. First, having an engaging personality could be the dominant feature in such individuals.[50] The mediator is similar with a symphony conductor, setting the tempo and guaranteeing proper entries for the ensemble cast,[51] to put fragment of narratives of the conflict together into euphony instead of cacophony. This leads to enthusiastic workplace communication that inspires others to be enthusiastic. Second, being patient is conducive to becoming effective at both teaching and learning. This training programme welcomed experienced professionals as well as beginners for not only lectures but also opinion exchanges. Senior and experienced seed teachers were expected to use their respective reserve talents to participate in the course and pass on their experiences to inexperienced personnel without difficulty. Patience is passion tamed. Those making good use of dynamics in both opposing and interdependent forces fit into the roles of mediators and would benefit from related training.

Limitations

Some limitations must be noted. First, the panel composition was imbalanced due to an uneven distribution of specialties. This might lead to overweighting the importance of medical staff and negligence of other specialties due to a false consensus effect, also known as consensus bias. Second, a retrospective follow-up to assess training results was lacking. To

For peer review only - http://bmjopen.bmj.com/site/about/guidelines.xhtml

understand the quality of the mediation service under current circumstances, establishment of a distinctive measurement tool with indicators for capabilities of perception, accountabilities, communication, and assistance is recommended. Third, a feedback mechanism should set up for the collection of opinions and suggestions from onboard mediators, specialists taking part in any step of the mediation or litigation, or our greenhorns to revise the curriculum. This syllabus could be considered a starting point, but further research is required to achieve perfection.

Conclusions

In today's society, people tend to confine themselves to limited roles and lack vision regarding a possible consequence of the fine division of labour. TIMEs is an essential component of multiparty relationships and establishing an organised model for skill cultivation was a major task. We recruited experienced specialists as moderators and members of the panel group and reached a consensus regarding the syllabus of the TIME programme, which comprised professionalism, core competences, and core curricula, after the four-round Delphi method was applied.

For peer review only

For peer review only - http://bmjopen.bmj.com/site/about/guidelines.xhtml

Abbreviations

ADR: alternative dispute resolution; IHM: In-house mediation; MOHW: Ministry of Health and Welfare; PCC: person-centred caring; SVS: second victim syndrome; TIME: triple-I mediation; TDRF: Taiwan Drug Relief Foundation

Declarations

Ethics approval and consent to participate

Experts from various fields were consulted to reach consensus regarding the syllabus of the TIME Training Programme by Taiwan's MOHW (Registration Number: MOHW1091110947). All participants signed an informed consent form, and the anonymity of personal information during data analysis was guaranteed. Ethical approval for data collection for this research was issued in accordance with paragraph 4, article 9 of the Personal Data Protection Act as per the protocol for academic research in the public interest in that data processed by the data provider or disclosed did not lead to the identification of the participants. The MOHW has provided limited access to data for formal documentation since December 2019.

Consent for publication

Not applicable

Availability of data and materials

The datasets used and/or analysed during the current study are available from the corresponding author on reasonable request.

For peer review only

Competing interests

The authors a declare that they have no known competing financial interests or personal relationships that could have appeared to influence the work reported in this paper.

Funding

This work was supported by Taiwan's Ministry of Health and Welfare on the basis of the 2018 Quality Improvement Programme in Medical Dispute Resolution [grant number M07A3401].

Authors' contributions

YCS contributed to literature review, plan execution, data collection and management, and manuscript draft preparation. RNS contributed to the study design, data collection and management, and assistance to the principal investigator in programme execution. WWC was the current president of Taiwan Drug Relief Foundation and contributed to policy advisory and study design. YPL and CLS contributed to policy advisory and information gathering to develop, support, and implement policies. CCW was the principal investigator and contributed to the study conceptualisation and design, data analysis evaluation, and programme execution. The final manuscript was read and approved by all authors.

Acknowledgements

The authors thank Prof. Che-Ming Yang for sharing their experiences and providing input regarding mediation during medical disputes and Yu-Ying Huang (Attorney at Law; Chairman of Taiwan Association of Harmony Medicare and Patient) for legal counselling.

For peer review only - http://bmjopen.bmj.com/site/about/guidelines.xhtml

The authors appreciate the contribution of specialists from the Taiwan Ministry of Health and Welfare in the integration of knowledge (Registration Number: MOHW1100023690). The authors also thank the Taiwan Drug Relief Foundation's project working group members, including Pin-Hsien Yeh, Hsin-Hsin Chen, and Po-Han Chen, for their contributions towards data collection and analysis and administrative support. We acknowledge the editor and series editor of *BMJ Open* for constructive criticism of an earlier version of this article.

For peer review only

Figure 1. Flowchart of the execution of the triple-I mediation (TIME) programme.

If a dispute case is referred for dispute consultation, mediators attend the relevant location and collaborate with their team members of different specialties depending on the scale and type of the dispute. As a fair and objective third party, they provide their services equally to both patients and medical staff. If a settlement is not reached, the dispute case is referred to a mediation committee at the department of health of the local city government for further case review and processing. If both medical staff and patients cannot reach a final agreement, the case is sent for a trial for a final decision to be made by a judge.

For peer review only

For peer review only - http://bmjopen.bmj.com/site/about/guidelines.xhtml

From cacophony to euphony: Time for Medical Dispute

Table 1

Characteristics of the panel members who completed the four-round Delphi survey

	Number (%)
Sex	
Male	7 (63.6)
Female	4 (36.4)
Age	
≥ 50 years	8 (72.7)
< 50 years	3 (27.3)
Occupation	
Physician	6 (54.5)
Lawyer	2 (18.2)
Judge	1 (9.1)
Clinical psychologist	1 (9.1)
Social worker	1 (9.1)
Experience with mediation, years	
≥ 10 years	11 (100)
< 10 years	0

For peer review only

From cacophony to euphony: Time for Medical Dispute

Table 2

Round 2 survey responses: core competences of mediators

	Strongly agree (n, %)	Agree (n, %)	Somewhat agree (n, %)	Neutral (n, %)	Somewhat disagree (n, %)	Disagree (n, %)	Strongly disagree (n, %)	Agreement (n, %)
Knowledge base of law	6 (54.5)	1 (9.1)	2 (18.2)	2 (18.2)	0 (0)	0 (0)	0 (0)	9 (81.2%)
Internalisation of the denotative and connotative meanings of care	6 (54.5)	4 (36.4)	1 (9.1)	0 (0)	0 (0)	0 (0)	0 (0)	11 (100%)
Effective, smooth, and timely communication	10 (90.9)	1 (9.1)	0 (0)	0 (0)	0 (0)	0 (0)	0 (0)	11 (100%)
Conflict resolution	6 (54.5)	5 (45.5)	0 (0)	0 (0)	0 (0)	0 (0)	0 (0)	11 (100%)

Table 3

Round 3 survey responses: core curricula of mediators

Core competence	Core curriculum	Strongly agree (n, %)	Agree (n, %)	Somewhat agree (n, %)	Neutral (n, %)	Somewhat disagree (n, %)	Disagree (n, %)	Strongly disagree (n, %)	Agreement (n, %)
Knowledge base of law	Overview of medical disputes and medical malpractice: current trends in Taiwan	4 (36.3)	5 (45.5)	2 (18.2)	0 (0)	0 (0)	0 (0)	0 (0)	11 (100%)
	Tort law dealing with professional negligence in medical malpractice and tort liability related to medical accidents: rights and responsibilities you should know	5 (45.5)	4 (36.3)	2 (18.2)	0 (0)	0 (0)	0 (0)	0 (0)	11 (100%)
	Contentious proceedings in a law case with medical errors: conventional means	3 (27.3)	3 (27.3)	4 (36.3)	1 (9.1)	0 (0)	0 (0)	0 (0)	90.9%

For peer review only - http://bmjopen.bmj.com/site/about/guidelines.xhtml

From cacophony to euphony: Time for Medical Dispute

	Current noncontentious approaches in dealing with dispute resolution: approaches involving tactical flexibility	7 (63.6)	4 (36.4)	0 (0)	0 (0)	0 (0)	0 (0)	0 (0)	11 (100%)
Internalisation of the denotative and connotative meanings of care	Core meanings of person-centred care: guidance involving holistic approach	9 (81.8)	2 (18.2)	0 (0)	0 (0)	0 (0)	0 (0)	0 (0)	11 (100%)
	Practical aspects of person-centred care: be straightforward without being rude	10 (90.9)	1 (9.1)	0 (0)	0 (0)	0 (0)	0 (0)	0 (0)	11 (100%)
	Duties and responsibilities of a triple-I health mediator: balancing obligation and liability	6 (54.5)	5 (45.5)	0 (0)	0 (0)	0 (0)	0 (0)	0 (0)	11 (100%)
	Nationwide accessibility of mediation services: barriers no longer exist among different levels of healthcare institutions	4 (36.4)	7 (63.6)	0 (0)	0 (0)	0 (0)	0 (0)	0 (0)	11 (100%)
	Integration of present-day resources: foundations dealing with drug relief, childbirth accident relief, damage compensation for injury, and programme for national vaccine injury compensation	4 (36.4)	4 (36.4)	3 (27.2)	0 (0)	0 (0)	0 (0)	0 (0)	11 (100%)
Effective, smooth, and timely communication	Ethics of a triple-I health mediator: knowing the difference between what you have a right to do and what is right to do	10 (90.9)	1 (9.1)	0 (0)	0 (0)	0 (0)	0 (0)	0 (0)	11 (100%)

For peer review only

For peer review only - http://bmjopen.bmj.com/site/about/guidelines.xhtml

From cacophony to euphony: Time for Medical Dispute

	Predecessors in the management of mediation: think ahead in time	9 (81.8)	2 (18.2)	0 (0)	0 (0)	0 (0)	0 (0)	0 (0)	11 (100%)
	Establishment of communication: an impregnable foundation leaving no boundary and obstacle	10 (90.9)	1 (9.1)	0 (0)	0 (0)	0 (0)	0 (0)	0 (0)	11 (100%)
	Acknowledge the needs and expectations of patients	8 (72.7)	3 (27.3)	0 (0)	0 (0)	0 (0)	0 (0)	0 (0)	11 (100%)
	Psychological analysis of patients: critical starting point of lean thinking	8 (72.7)	3 (27.3)	0 (0)	0 (0)	0 (0)	0 (0)	0 (0)	11 (100%)
	Grief counselling: reach out for help	7 (63.6)	4 (36.4)	0 (0)	0 (0)	0 (0)	0 (0)	0 (0)	11 (100%)
	Emotion recognition and corresponding responses: knowing me, knowing you	9 (81.8)	2 (18.2)	0 (0)	0 (0)	0 (0)	0 (0)	0 (0)	11 (100%)
Conflict resolution	Disparity in problem recognition between physicians and patients: gaps you cannot ignore	6 (54.5)	5 (45.5)	0 (0)	0 (0)	0 (0)	0 (0)	0 (0)	11 (100%)
	Strategies and tips for conflict resolution: learn the ropes in advance	9 (81.8)	2 (18.2)	0 (0)	0 (0)	0 (0)	0 (0)	0 (0)	11 (100%)
	Tactics and hints for facilitating effective communication: the bond-forming factor in all human relations is conversation	8 (72.7)	3 (27.3)	0 (0)	0 (0)	0 (0)	0 (0)	0 (0)	11 (100%)
	Frequently asked questions and answers: power-up your abilities without hesitation	10 (90.9)	1 (9.1)	0 (0)	0 (0)	0 (0)	0 (0)	0 (0)	11 (100%)

For peer review only

For peer review only - http://bmjopen.bmj.com/site/about/guidelines.xhtml

From cacophony to euphony: Time for Medical Dispute

Table 4

Final product of core curricula after a four-round Delphi survey

Curriculum	Duration	Description
Knowledge base of law	1 hour	(01) Overview of medical dispute and medical malpractice: currents trend in Taiwan<br>(02) Tort law dealing with professional negligence in medical malpractice and tort liability related to medical accidents: rights and responsibilities you should know<br>(03) Contentious proceedings in a law case with medical errors: conventional means<br>(04) Current noncontentious approaches in dealing with dispute resolution: approaches involving tactical flexibility
Internalisation of the denotative and connotative meanings of care	1 hour	(01) Core meanings of person-centred care: guidance involving holistic approach<br>(02) Practical aspects of person-centred care: be straightforward without being rude<br>(03) Duties and responsibilities of a triple-I health mediator: balancing obligation and liability<br>(04) Nationwide accessibility of mediation services: barriers no longer exist among different levels of healthcare institutions<br>(05) Integration of present-day resources: foundations dealing with drug relief, childbirth accident relief, damage compensation for injury, and programme for national vaccine injury compensations.
Effective, smooth, and timely communication (1-hour lectures, 2-hour practice)	3 hours	(01) Ethics of a triple-I health mediator: knowing the difference between what you have a right to do and what is right to do<br>(02) Predecessors in the management of mediation: think ahead in time<br>(03) Establishment of communication: an impregnable foundation leaving no boundary and obstacle<br>(04) Acknowledge the needs and expectations of patients<br>(05) Psychological analysis of patients: critical starting point of lean thinking<br>(06) Grief counselling: reach out for help<br>(07) Emotion recognition and corresponding responses: knowing me, knowing you
Conflict resolution (1-hour lectures, 2-hour practice)	3 hours	(01) Disparity in problem recognition between physicians and patients: gaps you cannot ignore<br>(02) Strategies and tips for conflict resolution: learn the ropes in advance<br>(03) Tactics and hints for facilitating effective communication: the bond-forming factor in all human relations is conversation<br>(04) Frequently asked questions and answers: power-up your abilities without hesitation

For peer review only - http://bmjopen.bmj.com/site/about/guidelines.xhtml

References:

1. Wu CY, Weng HC, Chen RC. Time trends of assessments for medical dispute cases in Taiwan: a 20-year nationwide study. *Intern Med J* 2013;43(9):1023-30.

2. Szmania SJ, Johnson AM, Mulligan M. Alternative dispute resolution in medical malpractice: A survey of emerging trends and practices. *Confl Resolut Q* 2008;26(1):71-96.

3. Sohn DH, Bal BS. Medical malpractice reform: the role of alternative dispute resolution. *Clin Orthop Relat Res* 2012;470(5):1370-8.

4. Wang M, Liu GG, Zhao H, et al. The role of mediation in solving medical disputes in China. *BMC Health Serv Res* 2020;20(1):225.

5. Tena-Tamayo C, Sotelo J. Malpractice in Mexico: arbitration not litigation. *Brit Med J* 2005;331(7514):448-51.

6. Giraldo P, Sato L, Martínez-Sánchez JM, et al. Eleven-year descriptive analysis of closed court verdicts on medical errors in Spain and Massachusetts. *BMJ Open* 2016;6(8):e011644-e.

7. Barbot J, Parizot I, Winance M. "No-fault" compensation for victims of medical injuries. Ten years of implementing the French model. *Health Policy* 2014;114(2-3):236-45.

8. Lee A, Bishop G, Hillman KM, et al. The Medical Emergency Team. *Anaesth Intensive Care Med* 1995;23(2):183-6.

9. Devita MA, Braithwaite RS, Mahidhara R, et al. Use of medical emergency team responses to reduce hospital cardiopulmonary arrests. *BMJ Qual Saf* 2004;13(4):251-4.

10. Winters BD, Weaver SJ, Pfoh ER, et al. Rapid-response systems as a patient safety strategy: a systematic review. *Ann Intern Med* 2013;158(5 Pt 2):417-25.

11. Murphy MK, Black NA, Lamping DL, et al. Consensus development methods, and their use in clinical guideline development. *Health Technol Assess* 1998;2(3):i-iv, 1-88.

For peer review only - http://bmjopen.bmj.com/site/about/guidelines.xhtml

12. Hasson F, Keeney S, Mckenna H. Research guidelines for the Delphi survey technique. *J Adv Nurs* 2000;32(4):1008-15.

13. Ogrinc G, Davies L, Goodman D, et al. SQUIRE 2.0 (Standards for QUality Improvement Reporting Excellence): revised publication guidelines from a detailed consensus process. *BMJ Qual Saf* 2016;25(12):986-92.

14. Chia-Chien H, Sandford B. The Delphi technique: making sense of consensus. *Pract Assess Res Evaluation* 2007;12(10).

15. Thelma S. The Delphi technique: an adaptive research tool. *Br J Occup Ther*;61(4):153-6.

16. Mueller PS. Incorporating professionalism into medical education: the Mayo Clinic experience. *Keio J Med* 2009;58(3):133-43.

17. Nursing_Directorate. Compassion in Practice Two years on Experience matters. NHS England; 2014.

18. Banka G, Edgington S, Kyulo N, et al. Improving patient satisfaction through physician education, feedback, and incentives. *J Hosp Med* 2015;10(8):497-502.

19. Table of cases reported annually. Taiwan: Ministry of Health and Welfare; 2021 [Available from: https://www.mohw.gov.tw/dl-67160-c1a18b5e-9da4-4d91-96a8-1fc87767f0fb.html.

20. Table of case type annually. Taiwan: Ministry of Health and Welfare; 2021 [Available from: https://www.mohw.gov.tw/dl-67161-b238a4c6-73c8-4eb5-976e-df9055141daf.html.

21. Table of factual test of causation annually Taiwan: Ministry of Health and Welfare; 2021 [Available from: https://www.mohw.gov.tw/dl-67162-0dff04eb-1f24-4853-b49c-ce4959ed16b6.html.

For peer review only

22. Gerardi D. Using mediation techniques to manage conflict and create healthy work environments. *AACN Clin Issues* 2004;15(2):182-95.

23. Saundry R, Mcardle L, Thomas P. Reframing workplace relations? Conflict resolution and mediation in a primary care trust. *Work Employ Soc* 2013;27(2):213-31.

24. Kosa K, Katona C, Papp M, et al. Health mediators as members of multidisciplinary group practice: lessons learned from a primary health care model programme in Hungary. *BMC Fam Pract* 2020;21(1):19.

25. Richards T. Chasms in communication. *Brit Med J* 1990;301:1407–8.

26. Studdert DM, Mello MM, Sage WM, et al. Defensive medicine among high-risk specialist physicians in a volatile malpractice environment. *Jama* 2005;293(21):2609-17.

27. Al Shamsi H, Almutairi AG, Al Mashrafi S, et al. Implications of Language Barriers for Healthcare: A Systematic Review. *Oman Med J* 2020;35(2):e122.

28. Jones S. Putting the Person into Person-Centered and Immediate Emotional Support. *Commun Res* 2016;31(3):338-60.

29. Ha JF, Longnecker N. Doctor-Patient Communication: A Review. *Ochsner J* 2010;10:38–43.

30. Lieberman E, Foux-Levy Y, Segal P. Beyond basic training: A model for developing mediator competence. *Confl Resolut Q* 2005;23(2):237-57.

31. Wegrzyn M, Vogt M, Kireclioglu B, et al. Mapping the emotional face. How individual face parts contribute to successful emotion recognition. *PLoS One* 2017;12(5):e0177239.

32. Stroebe M, Schut H, Boerner K. Cautioning Health-Care Professionals. *Omega (Westport)* 2017;74(4):455-73.

33. Brunjes GB. Spiritual pain and suffering. *Asian Pac J Cancer Prev* 2011;11(Suppl 1):31-6.

For peer review only

34. Koenig HG. Religion, spirituality, and health: the research and clinical implications. *ISRN Psychiatry* 2012;2012:278730.

35. Zajacova A, Lawrence EM. The Relationship Between Education and Health: Reducing Disparities Through a Contextual Approach. *Annu Rev Public Health* 2018;39:273-89.

36. Ohrnberger J, Fichera E, Sutton M. The relationship between physical and mental health: A mediation analysis. *Soc Sci Med* 2017;195:42-9.

37. Mcknight PE, Monfort SS, Kashdan TB, et al. Anxiety symptoms and functional impairment: A systematic review of the correlation between the two measures. *Clin Psychol Rev* 2016;45:115-30.

38. Mcknight PE, Kashdan TB. The importance of functional impairment to mental health outcomes: a case for reassessing our goals in depression treatment research. *Clin Psychol Rev* 2009;29(3):243-59.

39. Bush RAB, Folger JP. The Promise of Mediation: Responding to Conflict Through Empowerment and Recognition: San Francisco, CA: Jossey-Bass; 1994.

40. Sally Higginbotham BA. Transformative Mediation and Gestalt: The Good Form of Empowerment and Recognition: A Response to Lisa Gaynier. *Gestalt rev* 2003;7(3):204-9.

41. Epstein RM, Franks P, Fiscella K, et al. Measuring patient-centered communication in patient-physician consultations: theoretical and practical issues. *Soc Sci Med* 2005;61(7):1516-28.

42. Kumar S. Burnout and Doctors: Prevalence, Prevention and Intervention. *Healthcare (Basel)* 2016;4(3).

43. Lemaire JB, Wallace JE. Burnout among doctors. *Bmj* 2017;358:j3360.

44. Albert W W. Medical error: the second victim. The doctor who makes the mistake needs help too. *Brit Med J* 2000;320(7237):726-7.

For peer review only - http://bmjopen.bmj.com/site/about/guidelines.xhtml

45. Ullstrom S, Andreen Sachs M, Hansson J, et al. Suffering in silence: a qualitative study of second victims of adverse events. *BMJ Qual Saf* 2014;23(4):325-31.

46. Kerasidou A, Horn R. Making space for empathy: supporting doctors in the emotional labour of clinical care. *BMC Med Ethics* 2016;17:8.

47. Studdert DM, Brennan TA. No-fault compensation for medical injuries: the prospect for error prevention. *Jama* 2001;286(2):217-23.

48. Kaplan SH, Greenfield S, Ware JE, Jr. Assessing the effects of physician-patient interactions on the outcomes of chronic disease. *Med Care* 1989;27(3 Suppl):S110-27.

49. Park EK, Song M. Communication barriers perceived by older patients and nurses. *Int J Nurs Stud* 2005;42(2):159-66.

50. Harmon KMJ. The Effective Mediator. *J PROF ISS ENG ED PR* 2006;132(4):326-33.

51. Waldman EA. Identifying the Role of Social Norms in Mediation: A Multiple Model Approach. *Hastings Law J* 1997;48(4):703.

For peer review only

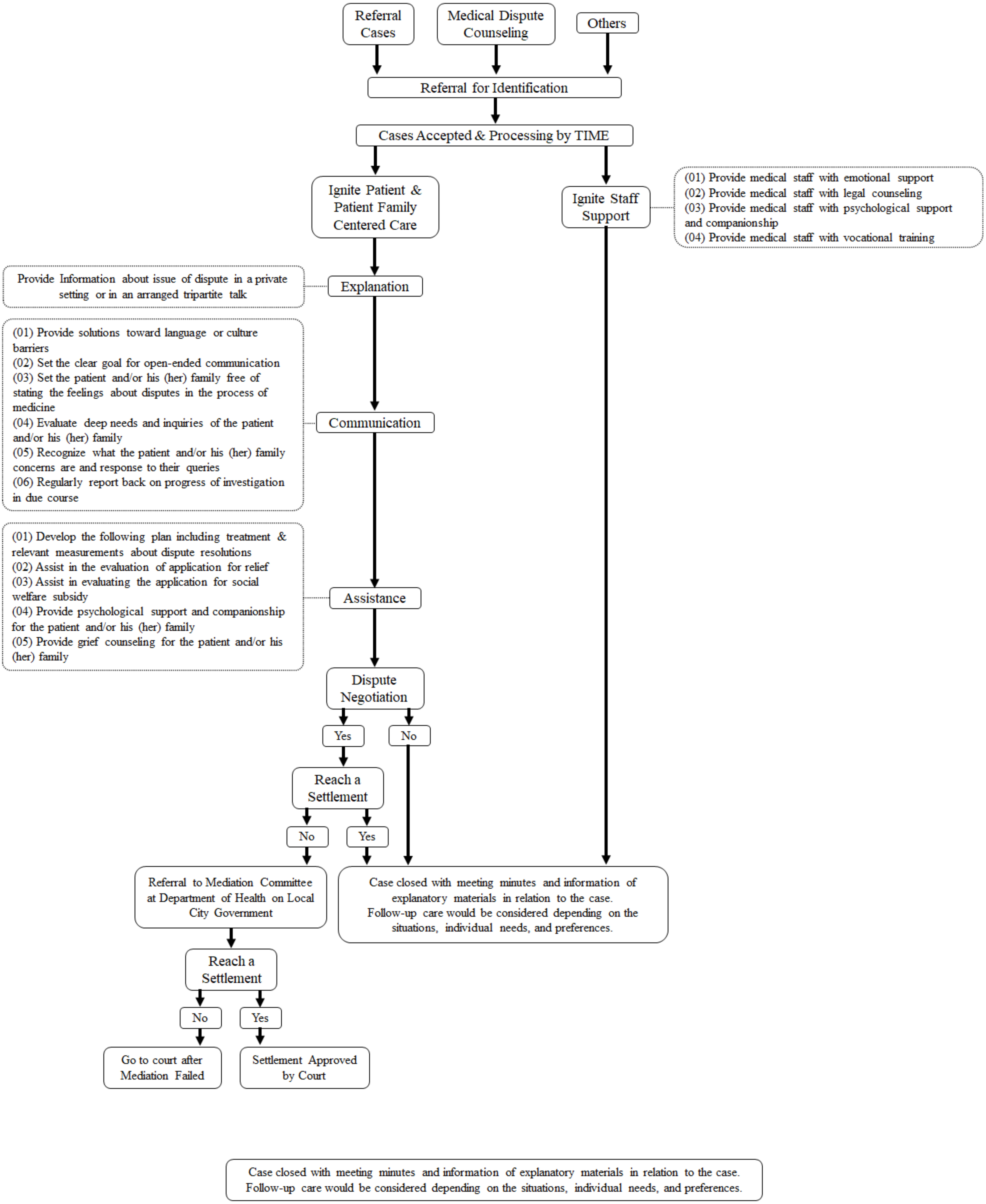

Figure 1. Flowchart of the execution of the triple-I mediation (TIME) programme.

If a dispute case is referred for dispute consultation, mediators attend the relevant location and collaborate with their team members of different specialties depending on the scale and type of the dispute. As a fair and objective third party, they provide their services equally to both patients and medical staff. If a settlement is not reached, the dispute case is referred to a mediation committee at the department of health of the local city government for further case review and processing. If both medical staff and patients cannot reach a final agreement, the case is sent for a trial for a final decision to be made by a judge.

Revised Standards for Quality Improvement Reporting Excellence (SQUIRE 2.0)
September 15, 2015

Text Section and Item Name	Section or Item Description	
**Notes to authors**	• The SQUIRE guidelines provide a framework for reporting new knowledge about how to improve healthcare<br><br>• The SQUIRE guidelines are intended for reports that describe system level work to improve the quality, safety, and value of healthcare, and used methods to establish that observed outcomes were due to the intervention(s).<br><br>• A range of approaches exists for improving healthcare. SQUIRE may be adapted for reporting any of these.<br><br>• Authors should consider every SQUIRE item, but it may be inappropriate or unnecessary to include every SQUIRE element in a particular manuscript.<br><br>• The SQUIRE Glossary contains definitions of many of the key words in SQUIRE.<br><br>• The Explanation and Elaboration document provides specific examples of well-written SQUIRE items, and an in-depth explanation of each item.<br><br>• Please cite SQUIRE when it is used to write a manuscript.	**As you review the manuscript, place a checkmark in this column for each SQUIRE item that is appropriately addressed in the manuscript. Remember that not every item is necessary in every manuscript.**
**Title and Abstract**		
**1. Title**	Indicate that the manuscript concerns an initiative to improve healthcare (broadly defined to include the quality, safety, effectiveness, patient-centeredness, timeliness, cost, efficiency, and equity of healthcare)	X
**2. Abstract**	a. Provide adequate information to aid in searching and indexing<br>b. Summarize all key information from various sections of the text using the abstract format of the intended publication or a structured summary such as: background, local problem, methods, interventions, results, conclusions	X

Introduction	*Why did you start?*	
3. Problem Description	Nature and significance of the local problem	X
4. Available knowledge	Summary of what is currently known about the problem, including relevant previous studies	X
5. Rationale	Informal or formal frameworks, models, concepts, and/or theories used to explain the problem, any reasons or assumptions that were used to develop the intervention(s), and reasons why the intervention(s) was expected to work	X
6. Specific aims	Purpose of the project and of this report	X
**Methods**	*What did you do?*	
7. Context	Contextual elements considered important at the outset of introducing the intervention(s)	X
8. Intervention(s)	a. Description of the intervention(s) in sufficient detail that others could reproduce it<br>b. Specifics of the team involved in the work	X
9. Study of the Intervention(s)	a. Approach chosen for assessing the impact of the intervention(s)<br>b. Approach used to establish whether the observed outcomes were due to the intervention(s)	X
10. Measures	a. Measures chosen for studying processes and outcomes of the intervention(s), including rationale for choosing them, their operational definitions, and their validity and reliability<br>b. Description of the approach to the ongoing assessment of contextual elements that contributed to the success, failure, efficiency, and cost<br>c. Methods employed for assessing completeness and accuracy of data	X
11. Analysis	a. Qualitative and quantitative methods used to draw inferences from the data<br>b. Methods for understanding variation within the data, including the effects of time as a variable	X
12. Ethical Considerations	Ethical aspects of implementing and studying the intervention(s) and how they were addressed, including, but not limited to, formal ethics review and potential conflict(s) of interest	X

For peer review only

**Results**	*What did you find?*	
**13. Results**	a. Initial steps of the intervention(s) and their evolution over time (*e.g.*, time-line diagram, flow chart, or table), including modifications made to the intervention during the project<br>b. Details of the process measures and outcome<br>c. Contextual elements that interacted with the intervention(s)<br>d. Observed associations between outcomes, interventions, and relevant contextual elements<br>e. Unintended consequences such as unexpected benefits, problems, failures, or costs associated with the intervention(s).<br>f. Details about missing data	X
**Discussion**	*What does it mean?*	
**14. Summary**	a. Key findings, including relevance to the rationale and specific aims<br>b. Particular strengths of the project	X
**15. Interpretation**	a. Nature of the association between the intervention(s) and the outcomes<br>b. Comparison of results with findings from other publications<br>c. Impact of the project on people and systems<br>d. Reasons for any differences between observed and anticipated outcomes, including the influence of context<br>e. Costs and strategic trade-offs, including opportunity costs	X
**16. Limitations**	a. Limits to the generalizability of the work<br>b. Factors that might have limited internal validity such as confounding, bias, or imprecision in the design, methods, measurement, or analysis<br>c. Efforts made to minimize and adjust for limitations	X
**17. Conclusions**	a. Usefulness of the work<br>b. Sustainability<br>c. Potential for spread to other contexts<br>d. Implications for practice and for further study in the field<br>e. Suggested next steps	X
**Other information**		
**18. Funding**	Sources of funding that supported this work. Role, if any, of the funding organization in the design, implementation, interpretation, and reporting	X

For peer review only

BMJ Open

The program of Triple-I mediator education (TIME) to improve medical disputes in clinical settings in Taiwan: a Delphi study

Journal:	*BMJ Open*
Manuscript ID	bmjopen-2021-058880.R1
Article Type:	Original research
Date Submitted by the Author:	06-Jul-2022

Complete List of Authors:	Shiao, Yi-Chih; Tri-Service General Hospital, Department of Family and Community Medicine; National Defense Medical Center, School of Medicine<br>Shen, Ruo-Nan; Taiwan Drug Relief Foundation<br>Chen, Wen-Wen; Taiwan Drug Relief Foundation<br>Liu, Yeh-Ping; Department of Medical Affairs; Taiwan Ministry of Health and Welfare<br>SHIH, Chung-Liang ; Taiwan Ministry of Health and Welfare<br>Wang, Chih-Chia; Tri-Service General Hospital, Department of Family and Community Medicine; National Defense Medical Center, School of Medicine

**Primary Subject Heading**:	Health policy
Secondary Subject Heading:	Health policy, Medical education and training
Keywords:	Health policy < HEALTH SERVICES ADMINISTRATION & MANAGEMENT, MEDICAL EDUCATION & TRAINING, PUBLIC HEALTH

SCHOLARONE™ Manuscripts

I, the Submitting Author has the right to grant and does grant on behalf of all authors of the Work (as defined in the below author licence), an exclusive licence and/or a non-exclusive licence for contributions from authors who are: i) UK Crown employees; ii) where BMJ has agreed a CC-BY licence shall apply, and/or iii) in accordance with the terms applicable for US Federal Government officers or employees acting as part of their official duties; on a worldwide, perpetual, irrevocable, royalty-free basis to BMJ Publishing Group Ltd ("BMJ") its licensees and where the relevant Journal is co-owned by BMJ to the co-owners of the Journal, to publish the Work in this journal and any other BMJ products and to exploit all rights, as set out in our licence.

The Submitting Author accepts and understands that any supply made under these terms is made by BMJ to the Submitting Author unless you are acting as an employee on behalf of your employer or a postgraduate student of an affiliated institution which is paying any applicable article publishing charge ("APC") for Open Access articles. Where the Submitting Author wishes to make the Work available on an Open Access basis (and intends to pay the relevant APC), the terms of reuse of such Open Access shall be governed by a Creative Commons licence – details of these licences and which Creative Commons licence will apply to this Work are set out in our licence referred to above.

Other than as permitted in any relevant BMJ Author's Self Archiving Policies, I confirm this Work has not been accepted for publication elsewhere, is not being considered for publication elsewhere and does not duplicate material already published. I confirm all authors consent to publication of this Work and authorise the granting of this licence.

For peer review only

The program of Triple-I mediator education (TIME) to improve medical disputes in clinical settings in Taiwan: a Delphi study

Yi-Chih Shiao[1,2,6], Ruo-Nan Shen[3], Wen-Wen Chen[3], Yueh-Ping Liu[4,5], Chung-Liang Shih[5], Chih-Chia Wang[1,2*]

*Correspondence: Chih-Chia Wang

Address: Number 325, Section 2, Chenggong Rd, Neihu District, 114, Taipei, Taiwan.

E-mail: tsghccwang@gmail.com

Tel: +886-2-87923311 ext. 16567

Fax: +886-2-87927057

[1]Department of Family and Community Medicine, Tri-Service General Hospital, National Defense Medical Center; No. 325, Section 2, Chenggong Rd, Neihu District, Taipei City 114, Taiwan (R.O.C.)

[2]School of Medicine, National Defense Medical Center, Taipei, Taiwan (R.O.C.); No. 161, Section 6, Minquan E Rd, Neihu District, Taipei City 114, Taiwan (R.O.C.)

[3]Taiwan Drug Relief Foundation, Taipei, Taiwan (R.O.C.); 10F., No. 22, Aiguo E. Rd., Zhongzheng Dist., Taipei City 100, Taiwan (R.O.C.)

[4]Department of Medical Affairs, Ministry of Health and Welfare, Taipei, Taiwan (R.O.C.); No. 488, Section 6, Zhongxiao E Rd, Nangang District, Taipei City 115, Taiwan (R.O.C.)

[5]Ministry of Health and Welfare, Taipei, Taiwan (R.O.C.); No. 488, Section 6, Zhongxiao E Rd, Nangang District, Taipei City 115, Taiwan (R.O.C.)

For peer review only

[6]College of Law, National Chengchi University, No.64, Sec.2, ZhiNan Rd., Wenshan District, Taipei City 11605, Taiwan.

Keywords: Alternative dispute resolution, Delphi method, in-house mediation, training programme syllabus

Manuscript word count: 3424 words

Abstract word count: 282 words

For peer review only

For peer review only - http://bmjopen.bmj.com/site/about/guidelines.xhtml

Abstract

Objectives To establish a training programme to cultivate trainee mediation skills through time investment, skill incorporation, and formation of in-house mediation services.

Design A four-round consensus conference was conducted by a number of seasoned experts selected in the manner of purposive sampling to determine core competences and relevant curricula through the modified Delphi process.

Setting Responses collected from enrolled experts through four rounds of the Delphi process from 11th November 2018 to 17th May 2019.

Participants Onboard seasoned mediators with different specialties.

Outcome measures Items with a median rating of 4 or more on a Likert scale of 1 to 7 points and 70% or more in agreement were identified as core competence and curricula.

Results Eleven enrolled experts reached the consensus about the training syllabus based on the 4-round agreement with four pillars of core competence, including 'knowledge base of law', 'internalisation of the denotative and connotative meanings of care', 'effective, smooth, and timely communication', and 'conflict resolution'. To grasp the dynamics and diversity of medical disputes on target, it is necessary to have sufficient knowledge and skills. We arrange our course in the order of teaching materials with pure didactics in the former two and with mixed contents comprising lectures and field exercises in the rest two.

Conclusions The sample developed a syllabus to train apprentices to take intermediate responses to medical disputes through the skills of conflict resolution and establishment of effective communication to improve the relationship between patients/relatives and medical staff, as a result of eventually reducing the conversion rate from dispute into litigation or alternative pathway. Policymakers in healthcare and top management in healthcare institutions can use this syllabus to guide their future education and training programme.

For peer review only - http://bmjopen.bmj.com/site/about/guidelines.xhtml

Strengths and limitations of this study

➢ The important strength of this study is the use of Delphi method providing anonymity to bring about detailed discussions.

➢ This study is the first official training program in Taiwan to train newcomers' skills in first-line mediation in clinical settings.

➢ This study is the beginning of a five-year Quality Improvement Program conducted by the Ministry of Healthcare and Welfare since 2018 and has the potential to be dedicated to policy improvement in fixing the broken and fragile relationships between medical personnel and patients/relatives.

➢ Limitations of our study include potentially selection bias caused by the method of purposive sampling and a lack of a follow-up system to assess training results.

For peer review only

For peer review only - http://bmjopen.bmj.com/site/about/guidelines.xhtml

Introduction

The number of medical disputes has been increasing[1]. An intermediate phase is the meaning of medical dispute between the preliminary status, which ‘conflict’ goes as a disagreement between medical staff and patients/relatives, and the dispute resolution status as the final stage. Most of these disputes belong to non-negligence after factual approach of causation in retrospective analysis all the time[2]. To resolve these disputes efficiently, several approaches has been utilized, including post-dispute problem-solving strategies with intra-juridical litigations and extra-juridical alternative dispute resolutions (ADRs) [3-5]. ADR has been integrated into the healthcare system of many countries[6-8] for mitigating risk of being sued and making both patients and physicians satisfied[3, 4]. Pre-dispute risk identification and early intervention, however, was merely merged into our current system.

As parts of dispute management, litigation and ADR, as post-dispute strategies, can be thought of as passive approaches compared with in-the-moment-of-dispute concerns from a third party independent from physicians and patients/relatives. In Taiwan, so-called active concerns about disputes are provided by a number of experts to fix the breakage in relationships between medical staff and patients/relatives and are dedicated to reducing the ratios of dispute-turning-into-litigation. These peace-bringing experts with different occupations, including physicians, lawyers, judge, psychologists, and social workers, are invited by the Ministry of Healthcare and Welfare (MOHW) based on their contributions, such as frontline mediators in hospitals, volunteer counselors in non-government organizations (NGOs) which are committed to the advocation of patient rights, and promoters of legal reform in the public and private sectors alike, so that our government can consult these experts when it encounters relevant disputes.

With the evolution of legislation, the draft act for the prevention and resolution of medical malpractice was proposed by the Executive Yuan in 2018. Under the legal

For peer review only - http://bmjopen.bmj.com/site/about/guidelines.xhtml

framework, active dispute care can alleviate the high tensions in doctor-patient relationships strained by medical disputes. A great demand for caring talents is anticipated, leading to the start of an associated programme named the triple-I mediator (TIME), authorized by the MOHW and characterized by ‘in-house argument handling’, ‘immediate action’, ‘integration resources’, to fix this talent gap. The triple-I mediator (TIME) program is the first formal training programme, born in response to address prevention of dispute turning into litigation on the legal basis of Article 82 of the Medical Care Art, and borrowed from the concept of medical emergency team[9, 10] or rapid response team[11] for the purpose of high acuity response to early deterioration of relationships between patients and physicians.

TIME, as the beginning of a five-year quality improvement series since 2018, a number of invited experts will attend a consensus meeting, conducted in a four-round Delphi process, for the extraction of experts’ experiences as the base of constructing a syllabus, including teaching knowledge and skills in the mediation field, to educate newcomers to become competent mediators. The study aims to identify the core competencies and confirm the teaching materials for future tutors to provide precise instructions to trainees.

For peer review only - http://bmjopen.bmj.com/site/about/guidelines.xhtml

Methods

Patient and public involvement

No patients and public were not involved in this study.

The Delphi method

The Delphi method is reliable and has been effectively used for establishing consensus regarding healthcare management[12]. It is a multistage, iterative, and anonymous process consisting of at least two rounds of surveys[13]. Opinions and feedback were structurally categorized under different headings after each round and exported to a temporary meeting document as reference to effectively facilitate discussions among moderators in the next round. Considering the critical paucity of relevant evidence for fostering skills, the Delphi method with four rounds of surveys was introduced to achieve consensus regarding the syllabus for the TIME programme. The strengths of this method included anonymity, capability of obtaining a collection of ideas and opinions from participants, and cost effectiveness. The weaknesses were the length of time required, research bias through the potential influence of moderators or participants, and uneven distribution of specialties among the respondents. The authors followed the guidance of Conducting and REporting DElphi Studies (CREDES)[14].

Agreement and consensus

Agreement was defined as the percentage of positive rather than neutral or negative responses divided by the total completed responses. Consensus was defined as 70% or more agreement according to the recommendations made by Thelma Sumsion[15].

The moderator of the TIME program

For peer review only

To integrate experts' opinions effectively, a clinical physician practicing family medicine with position as an associate professor in medicine and a Doctor of Laws degree, is designated as the moderator by the Taiwan Drug Relief Foundation (TDRF) due to his multiple experiences for interdisciplinary coordination, such as conducting a pilot project to encourage medical institutions to properly handle surgical and anaesthesia disputes in 2014 and bringing about the satisfactory outcome of the promotion of the Childbirth Accident Emergency Relief Act in 2015.

For peer review only

The sample of experts in the TIME program

Delbecq *et al* suggested that 10–15 members could be a satisfactory range for sample[16]. Our goal was to have at least 10 participants for each round. The experts participating in the TIME program were formally recruited in the manner of purposive sampling through granted consent of the expert invitation document sent by TDRF. These experts had more than 10 years of experience making sufficient contributions in the field of medical personnel-patient relationship repair, including serving as local civil mediators in various courts affiliated to the Judicial Yuan, and non-government organizations (NGOs) that provided legal services to patients/family members. Although the number of physicians outweighed other types of profession, it did not mean they stand the physicians' point of view. Instead, two of them long-term provided mental support and counselling to patients who were stuck in a poor relationship with their medical healthcare providers. In order to make sure a whole coverage of experts' opinions, the common triad encountered in medical disputes, including the arm of medical personnel, of patients/relatives, and the arm of legal professionals, were included in our study.

Rapid reviews

For peer review only - http://bmjopen.bmj.com/site/about/guidelines.xhtml

'Rapid reviews' is a useful tool accepted by the World Health Organization (WHO) to furnish evidence in the target field in a cost-effective fashion[17]. References with respect to dispute resolving and relevant training programs, reviewed by the moderator, would be provided to the participants in the first Delphi round. The spectrum of keywords would be set up as wide as possible to include enough publications in the field of training courses for resolving medical disputes (i.e., 'medical dispute'. 'conflict' and 'training'). Articles published between 1998 and 2018, written in English, and indexed in common databases, such as PubMed, Medline, and Embase, would be included. The sample considered whether they exhibited pillars of professionalism in the TIME programme based on relevant content in medical education[18], Roach's Six C's in caring[19], and a communication model called CI-CARE proposed by the University of California, Los Angeles Health System[20], as a benchmark. Furthermore, evidence unveiled mediation training could facilitate healthcare staff to resolve conflicts at an early stage[21]. The basics of training should include several aspects of conflicts, comprising the identification of conflict resources, application of conflict management, and upgrading nonverbal communication skills[22]. A follow-up interval of six months after the training program showed that the trainees became more competent in coping with conflicts[23].

Delphi survey rounds

In round 1, the sample was required to read aforementioned references reviewed by the moderator and provided details of their occupations, experience with regard to dealing with medical disputes in clinical settings, knowledge of conflict management, and coping strategies used to alleviate the high tensions between medical personnel and patients/relatives. Opinions and details on constructing core competences correlated with fostering professionalism and reflection after reading references were provided using

For peer review only - http://bmjopen.bmj.com/site/about/guidelines.xhtml

narrative text responses and discussion. The feedback and views of experts were carefully gathered to generate a draft questionnaire.

In round 2, the draft proposal comprising several features of core competences was presented and measured through a questionnaire answered using a 7-point Likert-type scale (1 = *strongly disagree*, 2 = *disagree*, 3 = *somewhat disagree*, 4 = *neutral*, 5 = *somewhat* agree, 6 = *agree*, and 7 = *strongly* agree). Consensus was based on an agreement level of >70%. Items with <70% agreement and relevant feedback regarding disagreement with a core competence was discussed by the moderator group. Participants were then requested to share opinions and details for constructing the core curricula correlating to fostering core competences by using narrative text responses. Information on positive and negative aspects mentioned earlier were gathered systematically to generate the initial draft containing the core competences and core curricula of the TIME programme.

In round 3, agreement among the panel members regarding the draft of the core curricula was obtained using a 7-point Likert scale similar to that used in round 2 with respect to the level of agreement by the respondents. Consensus was reached and discussions were held by the moderator group if any item had <70% agreement. Additional narrative text was provided to determine whether any disagreement existed regarding the core curriculum. This draft was revised by the group based on the analysis of agreement and annotation of round 3. Besides, participants were asked to calculate the time in each segment of this training program in self-simulated situations and the scheduled class time would be discussed in the fourth round.

In round 4, respondents were asked to provide suggestions on syntactic errors, improper phrasing, or terminology to make the description concise. The final version of the TIME training syllabus included (1) immediate action (2) policy incorporation (3) in-house settlement based on agreement in rounds 2 and 3. In regard to the class time of each segment,

For peer review only

including ‘Knowledge base of law’, ‘Internalisation of the denotative and connotative meanings of care’ , ‘Effective, smooth, and timely communication’, ‘Conflict resolution’, would be one, one, three, and three hours, respectively, after the participants reached the consensus after taking the reasonable length of content in each segment of this course into accounts. The final version of the syllabus draft was validated and announced on 15 November 2019, the promulgation date (Figure 1), accompanied with the announcement of the initial version of the official course handbook based on the draft by the MOHW. The latest version on the official website of the MOHW was free to the public with open access[24].

For peer review only

Results

The characteristics of the participants are presented in Table 1. All the 11 participants completed the four-round Delphi survey. The expert panel group consisted of 7 men (63.6%) and 4 women (36.4%). Physicians predominated the expert panel group (54.5 %). The respondents enrolled were experienced, knowledgeable, and skilful specialists of different fields possessing more than 10 -year experience.

Table 2 shows a consensus on all items, with more than 70% agreement. The item ‘effective, smooth, and timely communication’ had the highest number of responses of ‘strongly agree’ (n = 10, 90.9%), and the rest of the items, including ‘knowledge base of law’, ‘internalisation of the denotative and connotative meanings of care’, and ‘conflict resolution’, had almost equal agreement (n = 6, 54.5%). Only the item ‘knowledge base of the law’ elicited a neutral opinion (n = 2, 18.2%).

In table 3, a consensus was reached for all items, with an agreement of 100%, except for the item ‘proceeding in law case with medical disputes: conventional means’, which had 91.7% agreement. Only in the ‘knowledge base of law’ category was the number of participants who responded of strongly agree below 10 (n = 9). The other three categories had at least one item with >10 people indicating strong agreement. Additionally, in the categories of ‘effective, smooth, and timely communication’ and ‘conflict resolution’, consensus was reached on all of the correlated core curricula, with more than half of the members in strong agreement. Furthermore, only the category of ‘effective, smooth, and timely communication’ had two items for which 10 members were in strong agreement, namely ‘ethics of TIME: knowing the difference between what you have a right to do and what is right to do’ and ‘Establishment of communication: an impregnable foundation leaving no boundary and obstacle.

For peer review only

Discussion

After a four-round Delphi process was applied, consensus was reached regarding the syllabus for the TIME training programme, which was established by a panel consisting of highly experienced experts in mediation. Table 4 was the final product containing four items on core competencies with respect to relevant core curricula. Each item received at least 70% agreement, which represents convergence of opinions.

To optimise the present healthcare system, Taiwan's MOHW introduced an in-house mediation (IHM) system for conflict management in 2013[25]. According to current regulations on IHM in Taiwan, mediators are defined as sentinel and people with an engaging personality[26] who provide mediation for a suspected, gradually formed, or established dispute, and collaborate with team members from different specialties depending on the scale or type of dispute. The mediator is similar with a symphony conductor, setting the tempo and guaranteeing proper entries for the ensemble cast[27], to put fragment of narratives of the conflict together into euphony instead of cacophony. Of the total number of members in the team, at least three must be in-house medical staff in addition to the original teams of healthcare providers and nonmedical staff (e.g., legal professionals or social workers) and nonmedical staff should comprise at least one third of the team in line with law. The design of IHM is aimed at creating a 360° feedback mechanism and obtaining good understanding of excellence; in reality, the ultimate goal of IHM application in dispute resolution is to ensure highly effective teamwork for appropriate dispute resolution rather than blame shifting.

The quality of communication can determine the direction of the relationships between medical staff-patients/relatives. Poor communication stems from the gap in mutual understanding, moving to incredulity[28], and causing the overuse of defensive medicine as a non-violent strategy in the management of medical disputes[29]. This psychological chain of reaction could be fixed from the technical perspective of resolution management, such as

For peer review only - http://bmjopen.bmj.com/site/about/guidelines.xhtml

using Google Translate, a famous applied science-oriented technology, helping overcome language barriers and improve satisfaction levels among healthcare providers and patients[30]. The design of ideal mediators after our TIME training is not focused on technical aspects mentioned before but on in-person communication by arriving at the scene of the dispute immediately and providing immediate care. In hospital settings, a mediator can activate the integration of resources after engaging with both parties of a dispute. In clinical settings, patients focus on discomfort and ignore or miss relevant information regarding available treatments. The utility of this related mediation may be time sensitive. Mediators accelerate the use of individualised services targeting specific demands after the overall evaluation of case needs and concerns. For those with legal liability for any damage they cause, legal remedies affirm the validity of their claims and requests. Damage caused by those with no liability could be dealt with through compensation[31]. Common access points to humanitarian relief include Taiwan's drug injury relief system, compensation for vaccine injuries, and childbirth accident relief.

The core concept conveyed in the segment 'Effective, smooth, and timely communication' is that the quality of care would be maintained by the high-quality of patient-centred caring (PCC)[32, 33]. As the first step of PCC, demonstrating empathy, including verbal and non-verbal (e.g., body expressions[34]) clues in every visit, is a critical and teachable multi-phrase skill[35]. Six keys are taught in our course to facilitate effective empathy[36], including (1) discerning existence of emotions in the clinical setting, (2) taking a pause to imagine what the interviewer (e.g., medical personnel or patients/relatives) may think about, (3) stating mediators' perception of the interviewer's feeling (i.e., "It sounds like this event really frustrates you in depth …"), (4) making the stated feeling presented by the interviewer plausible, (5) showing respect for the interviewer's effort to tackle the dilemma, and (6) furnishing mental support and partnership (i.e., "Let's move to the action that we can

For peer review only - http://bmjopen.bmj.com/site/about/guidelines.xhtml

take together to...”). The trainers would teach trainees a formula comprising queries- clarifications- in order to memorize and to practice[37].

Trainees would learn the ropes of integrating emotional clues about physical or mental health from tutors in our teaching program. These collected threads will be the base for approaching the interviewee’s involved in medical dispute authentic feelings. To be more specific, detected emotions could be approximately categorized in the Kübler-Ross Change Curve (KRCC), including grief denial, anger, bargaining, depression, acceptance, and coping strategies could be conducted according to individual differences. However, one thing should be kept in mind that this category is just an auxiliary measure for approaching the interviewer’s authentic feelings. Stiffly putting someone’s fragment of emotional journey into the framework of the KRCC is inappropriate[38]. These detections of emotions by a well-educated mediator facilitate precise and rapid responses to such feelings because physical discomfort leads to mental and emotional suffering[39]. As physical health decline, either temporarily or permanently, would have mutual[40] and detrimental effects on psychological health (e.g., anxiety[41], depression[42]), leading to eventually undermining the existing/established-in-the-process partnerships and difficulty in building trust with others[43]. The two-hour practice in this segment of the TIME training program is designed to allow trainees to be proficient in providing emotional support through situational exercise and to receive verbal feedback on their performances by trainers.

When a medical dispute occurs, both medical personnel[44] and patients/relatives[45] would be influenced and become vulnerable, sensitive, and self-absorbed. The defense mechanism will be activated in both parties and cause the relationship to become increasingly alienated and destructive, and make people more injured, bringing about a form of vicious cycle. To break this cycle and restore the broken trust, the so-called ‘transformative mediation[46]’ with two key features is fundamental and would be practiced in the practicing

For peer review only - http://bmjopen.bmj.com/site/about/guidelines.xhtml

part of the course section ‘Conflict resolution’. One is empowerment shift[47], which could allow both parties to regain respect, confidence, and empathy. The other is recognition shift[47], which could help patients reduce their defensiveness against medical staff and gain a different perspective. This approach made patients express needs and strengthened bilateral participation in health care issue[48].

As we focused on the arm of patients/relatives, there would be ignorance of the need for care from the arm of medical personnel. This constantly neglected blind spot of calling for help for healthcare professions is going to be smoothened by mediators after the TIME training. The potential emotional trauma of staff in response to adverse patient responses can cause burnout[49]. Such burnout has been thought to be high among physicians globally[50]. Con-sequences of burnout have detrimental effects on doctors, which may lead to them making incorrect decisions (thus causing medical errors), exhibiting a hostile attitude towards patients, and having dysfunctional relationships with colleagues[49]. Once staff having traumatic experiences they would suffer from second victim syndrome (SVS)[51]. Mediators after the TIME training could recognize the six phrases, including the early stage of ‘initial chaos and accident response’ and the final of ‘moving on’[52]. In half of the process during the SVS recovery trajectory, especially in phrase three, the suffering personnel would try to seek help and re-establish trust with surroundings[52]. This would be the timing on target for mediators to approach involved personnel by listening empathetically, eschew judgement, and showing respect[53]. SVS cannot be treated if systematic and well-structured support for coping with emotional burdens is absent[54]. To put it in a nutshell, medical staff should be provided with adequate care similar to support for patients/relatives with mature, intact, and positive counselling[55].

Some limitations must be noted. First, the sample composition was imbalanced due to an uneven distribution of specialties caused by the method of purposive sampling. Doubts arise

For peer review only - http://bmjopen.bmj.com/site/about/guidelines.xhtml

as any possibility of selection bias still left. Second, a retrospective follow-up to assess training results was lacking. To understand the quality of the mediation service under current circumstances, establishment of a distinctive measurement tool with indicators for capabilities of perception, accountabilities, communication, and assistance is recommended. Third, a feedback mechanism should set up for the collection of opinions and suggestions from onboard mediators, specialists taking part in any step of the mediation or litigation, or our greenhorns to revise the curriculum. This syllabus could be considered a starting point, but further research is required to achieve perfection.

Conclusions

The TIME syllabus is the first official training program of mediators for medical disputes in Taiwan and is constructed after a four-round Delphi process with four sections, including ‘Knowledge base of law', ‘Internalisation of the denotative and connotative meanings of care', ‘Effective, smooth, and timely communication', and ‘Conflict resolution'. The design of the TIME syllabus, which contains lectures and field exercises for trainees to enrich their knowledge in relation to mediation and sharpen their skills in establishing firm relationships with medical personnel and/or patients/relatives and managing medical disputes.

For peer review only

Abbreviations

ADR: alternative dispute resolution; IHM: In-house mediation; MOHW: Ministry of Health and Welfare; PCC: person-centred caring; SVS: second victim syndrome; TIME: triple-I mediation; TDRF: Taiwan Drug Relief Foundation

Declarations

Ethics approval and consent to participate

The TIME program was approved by the Institutional Review Board (IRB) and the Research Ethics Committee of the Tri-Service General Hospital (TSGHIRB: E202216022) and was conducted in line with the Declaration of Helsinki. Informed consent was obtained along with the participants' responses.

Consent for publication

Not applicable

Availability of data and materials

The datasets used and/or analysed during the current study are available from the corresponding author on reasonable request.

Competing interests

The authors a declare that they have no known competing financial interests or personal relationships that could have appeared to influence the work reported in this paper.

Funding

For peer review only

This work was supported by Taiwan's Ministry of Health and Welfare on the basis of the 2018 Quality Improvement Programme in Medical Dispute Resolution [grant number: M07A3401].

Authors' contributions

YCS contributed to literature review, plan execution, data collection and management, and manuscript draft preparation. RNS contributed to the study design, data collection and management, and assistance to the principal investigator in programme execution. WWC was the current president of Taiwan Drug Relief Foundation and contributed to policy advisory and study design. YPL and CLS contributed to policy advisory and information gathering to develop, support, and implement policies. CCW was the principal investigator and contributed to the study conceptualisation and design, data analysis evaluation, and programme execution. The final manuscript was read and approved by all authors.

Acknowledgements

The authors thank Prof. Che-Ming Yang for sharing their experiences and providing input regarding mediation during medical disputes and Yu-Ying Huang (Attorney at Law; Chairman of Taiwan Association of Harmony Medicare and Patient) for legal counselling. The authors appreciate the contribution of specialists from the Taiwan Ministry of Health and Welfare in the integration of knowledge (Registration Number: MOHW1100023690). The authors also thank the Taiwan Drug Relief Foundation's project working group members, including Pin-Hsien Yeh, Hsin-Hsin Chen, and Po-Han Chen, for their contributions towards data collection and analysis and administrative support. We acknowledge the editor and series editor of *BMJ Open* for constructive criticism of an earlier version of this article.

For peer review only

For peer review only

For peer review only - http://bmjopen.bmj.com/site/about/guidelines.xhtml

Figure 1. Flowchart of the execution of the triple-I mediation (TIME) programme.

If a dispute case is referred for dispute consultation, mediators attend the relevant location and collaborate with their team members of different specialties depending on the scale and type of the dispute. As a fair and objective third party, they provide their services equally to both patients and medical staff. If a settlement is not reached, the dispute case is referred to a mediation committee at the department of health of the local city government for further case review and processing. If both medical staff and patients cannot reach a final agreement, the case is sent for a trial for a final decision to be made by a judge.

For peer review only

Table 1

Characteristics of the panel members who completed the four-round Delphi survey

	Number (%)
Sex	
Male	7 (63.6)
Female	4 (36.4)
Age	
≥ 50 years	8 (72.7)
< 50 years	3 (27.3)
Occupation	
Physician	6 (54.5)
Lawyer	2 (18.2)
Judge	1 (9.1)
Clinical psychologist	1 (9.1)
Social worker	1 (9.1)
Experience with mediation, years	
≥ 10 years	11 (100)
< 10 years	0

For peer review only

Table 2

Round 2 survey responses: core competences of mediators

	Strongly agree (n, %)	Agree (n, %)	Somewhat agree (n, %)	Neutral (n, %)	Somewhat disagree (n, %)	Disagree (n, %)	Strongly disagree (n, %)	Agreement (n, %)
Knowledge base of law	6 (54.5)	1 (9.1)	2 (18.2)	2 (18.2)	0 (0)	0 (0)	0 (0)	9 (81.2%)
Internalisation of the denotative and connotative meanings of care	6 (54.5)	4 (36.4)	1 (9.1)	0 (0)	0 (0)	0 (0)	0 (0)	11 (100%)
Effective, smooth, and timely communication	10 (90.9)	1 (9.1)	0 (0)	0 (0)	0 (0)	0 (0)	0 (0)	11 (100%)
Conflict resolution	6 (54.5)	5 (45.5)	0 (0)	0 (0)	0 (0)	0 (0)	0 (0)	11 (100%)

Table 3

Round 3 survey responses: core curricula of mediators

Core competence	Core curriculum	Strongly agree (n, %)	Agree (n, %)	Somewhat agree (n, %)	Neutral (n, %)	Somewhat disagree (n, %)	Disagree (n, %)	Strongly disagree (n, %)	Agreement (n, %)
Knowledge base of law	Overview of medical disputes and medical malpractice: current trends in Taiwan	4 (36.3)	5 (45.5)	2 (18.2)	0 (0)	0 (0)	0 (0)	0 (0)	11 (100%)
	Tort law dealing with professional negligence in medical malpractice and tort liability related to medical accidents: rights and responsibilities you should know	5 (45.5)	4 (36.3)	2 (18.2)	0 (0)	0 (0)	0 (0)	0 (0)	11 (100%)
	Proceedings in a law case with medical disputes: conventional means	3 (27.3)	3 (27.3)	4 (36.3)	1 (9.1)	0 (0)	0 (0)	0 (0)	90.9%

For peer review only - http://bmjopen.bmj.com/site/about/guidelines.xhtml

	Current noncontentious approaches in dealing with dispute resolution: approaches involving tactical flexibility	7 (63.6)	4 (36.4)	0 (0)	0 (0)	0 (0)	0 (0)	0 (0)	11 (100%)
Internalisation of the denotative and connotative meanings of care	Core meanings of person-centred care: guidance involving holistic approach	9 (81.8)	2 (18.2)	0 (0)	0 (0)	0 (0)	0 (0)	0 (0)	11 (100%)
	Practical aspects of person-centred care: be straightforward without being rude	10 (90.9)	1 (9.1)	0 (0)	0 (0)	0 (0)	0 (0)	0 (0)	11 (100%)
	Duties and responsibilities of a triple-I health mediator: balancing obligation and liability	6 (54.5)	5 (45.5)	0 (0)	0 (0)	0 (0)	0 (0)	0 (0)	11 (100%)
	Nationwide accessibility of mediation services: barriers no longer exist among different levels of healthcare institutions	4 (36.4)	7 (63.6)	0 (0)	0 (0)	0 (0)	0 (0)	0 (0)	11 (100%)
	Integration of present-day resources: foundations dealing with drug relief, childbirth accident relief, damage compensation for injury, and programme for national vaccine injury compensation	4 (36.4)	4 (36.4)	3 (27.2)	0 (0)	0 (0)	0 (0)	0 (0)	11 (100%)
Effective, smooth, and timely communication	Ethics of a triple-I health mediator: knowing the difference between what you have a right to do and what is right to do	10 (90.9)	1 (9.1)	0 (0)	0 (0)	0 (0)	0 (0)	0 (0)	11 (100%)
	Predecessors in the management of mediation: think ahead in time	9 (81.8)	2 (18.2)	0 (0)	0 (0)	0 (0)	0 (0)	0 (0)	11 (100%)

For peer review only

	Establishment of communication: an impregnable foundation leaving no boundary and obstacle	10 (90.9)	1 (9.1)	0 (0)	0 (0)	0 (0)	0 (0)	0 (0)	11 (100%)
	Acknowledge the needs and expectations of patients/relatives and medical personnel	8 (72.7)	3 (27.3)	0 (0)	0 (0)	0 (0)	0 (0)	0 (0)	11 (100%)
	Psychological analysis of patients: critical starting point of lean thinking	8 (72.7)	3 (27.3)	0 (0)	0 (0)	0 (0)	0 (0)	0 (0)	11 (100%)
	Grief counselling: reach out for help	7 (63.6)	4 (36.4)	0 (0)	0 (0)	0 (0)	0 (0)	0 (0)	11 (100%)
	Emotion recognition and corresponding responses: knowing me, knowing you	9 (81.8)	2 (18.2)	0 (0)	0 (0)	0 (0)	0 (0)	0 (0)	11 (100%)
Conflict resolution	Disparity in problem recognition between physicians and patients: gaps you cannot ignore	6 (54.5)	5 (45.5)	0 (0)	0 (0)	0 (0)	0 (0)	0 (0)	11 (100%)
	Strategies and tips for conflict resolution: learn the ropes in advance	9 (81.8)	2 (18.2)	0 (0)	0 (0)	0 (0)	0 (0)	0 (0)	11 (100%)
	Tactics and hints for facilitating effective communication: the bond-forming factor in all human relations is conversation	8 (72.7)	3 (27.3)	0 (0)	0 (0)	0 (0)	0 (0)	0 (0)	11 (100%)
	Frequently asked questions and answers: power-up your abilities without hesitation	10 (90.9)	1 (9.1)	0 (0)	0 (0)	0 (0)	0 (0)	0 (0)	11 (100%)

For peer review only - http://bmjopen.bmj.com/site/about/guidelines.xhtml

Table 4

Final product of core curricula after a four-round Delphi survey

Curriculum	Duration	Description
Knowledge base of law	1 hour	(01) Overview of medical dispute and medical malpractice: currents trend in Taiwan<br>(02) Tort law dealing with professional negligence in medical malpractice and tort liability related to medical accidents: rights and responsibilities you should know<br>(03) Proceedings in a law case with medical disputes: conventional means<br>(04) Current noncontentious approaches in dealing with dispute resolution: approaches involving tactical flexibility
Internalisation of the denotative and connotative meanings of care	1 hour	(01) Core meanings of person-centred care: guidance involving holistic approach<br>(02) Practical aspects of person-centred care: be straightforward without being rude<br>(03) Duties and responsibilities of a triple-I health mediator: balancing obligation and liability<br>(04) Nationwide accessibility of mediation services: barriers no longer exist among different levels of healthcare institutions<br>(05) Integration of present-day resources: foundations dealing with drug relief, childbirth accident relief, damage compensation for injury, and programme for national vaccine injury compensations.
Effective, smooth, and timely communication (1-hour lectures, 2-hour practice)	3 hours	(01) Ethics of a triple-I health mediator: knowing the difference between what you have a right to do and what is right to do<br>(02) Predecessors in the management of mediation: think ahead in time<br>(03) Establishment of communication: an impregnable foundation leaving no boundary and obstacle<br>(04) Acknowledge the needs and expectations of patients/relatives and medical personnel<br>(05) Psychological analysis of patients: critical starting point of lean thinking<br>(06) Grief counselling: reach out for help<br>(07) Emotion recognition and corresponding responses: knowing me, knowing you
Conflict resolution (1-hour lectures, 2-hour practice)	3 hours	(01) Disparity in problem recognition between physicians and patients: gaps you cannot ignore<br>(02) Strategies and tips for conflict resolution: learn the ropes in advance<br>(03) Tactics and hints for facilitating effective communication: the bond-forming factor in all human relations is conversation<br>(04) Frequently asked questions and answers: power-up your abilities without hesitation

For peer review only

References:

1. Wu CY, Weng HC, Chen RC. Time trends of assessments for medical dispute cases in Taiwan: a 20-year nationwide study. *Intern Med J* 2013;43(9):1023-30.

2. Table of factual test of causation annually Taiwan: Ministry of Health and Welfare; 2021. Available from: https://www.mohw.gov.tw/dl-67162-0dff04eb-1f24-4853-b49c-ce4959ed16b6.html. (accessed 5 August 2021).

3. Szmania SJ, Johnson AM, Mulligan M. Alternative dispute resolution in medical malpractice: A survey of emerging trends and practices. *Confl Resolut Q* 2008;26(1):71-96.

4. Sohn DH, Bal BS. Medical malpractice reform: the role of alternative dispute resolution. *Clin Orthop Relat Res* 2012;470(5):1370-8.

5. Wang M, Liu GG, Zhao H, et al. The role of mediation in solving medical disputes in China. *BMC Health Serv Res* 2020;20(1):225.

6. Tena-Tamayo C, Sotelo J. Malpractice in Mexico: arbitration not litigation. *Brit Med J* 2005;331(7514):448-51.

7. Giraldo P, Sato L, Martínez-Sánchez JM, et al. Eleven-year descriptive analysis of closed court verdicts on medical errors in Spain and Massachusetts. *BMJ Open* 2016;6(8):e011644-e.

8. Barbot J, Parizot I, Winance M. "No-fault" compensation for victims of medical injuries. Ten years of implementing the French model. *Health Policy* 2014;114(2-3):236-45.

9. Lee A, Bishop G, Hillman KM, et al. The Medical Emergency Team. *Anaesth Intensive Care Med* 1995;23(2):183-6.

10. Devita MA, Braithwaite RS, Mahidhara R, et al. Use of medical emergency team responses to reduce hospital cardiopulmonary arrests. *BMJ Qual Saf* 2004;13(4):251-4.

11. Winters BD, Weaver SJ, Pfoh ER, et al. Rapid-response systems as a patient safety strategy: a systematic review. *Ann Intern Med* 2013;158(5 Pt 2):417-25.

For peer review only

12. Murphy MK, Black NA, Lamping DL, et al. Consensus development methods, and their use in clinical guideline development. *Health Technol Assess* 1998;2(3):i-iv, 1-88.

13. Hasson F, Keeney S, Mckenna H. Research guidelines for the Delphi survey technique. *J Adv Nurs* 2000;32(4):1008-15.

14. Jünger S, Payne SA, Brine J, et al. Guidance on Conducting and REporting DElphi Studies (CREDES) in palliative care: Recommendations based on a methodological systematic review. *Palliat Med* 2017;31(8):684-706.

15. Sumsion T. The Delphi technique: an adaptive research tool. *Br J Occup Ther*;61(4):153-6.

16. Delbecq A, Ven A, Gustafson D. Group Techniques for Program Planning: A Guide to Nominal Group and Delphi Processes. Glenview, Illinois: Scott Foresman and Company; 1975.

17. Tricco AC, Langlois E, Straus SE, et al. Rapid reviews to strengthen health policy and systems: a practical guide: World Health Organization; 2017.

18. Mueller PS. Incorporating professionalism into medical education: the Mayo Clinic experience. *Keio J Med* 2009;58(3):133-43.

19. Nursing_Directorate. Compassion in Practice Two years on Experience matters. NHS England; 2014.

20. Banka G, Edgington S, Kyulo N, et al. Improving patient satisfaction through physician education, feedback, and incentives. *J Hosp Med* 2015;10(8):497-502.

21. Saulo M, Wagener RJ. Mediation training enhances conflict management by healthcare personnel. *Am J Manag Care* 2000;6(4):473-83.

22. Wolfe AD, Hoang KB, Denniston SF. Teaching Conflict Resolution in Medicine: Lessons From Business, Diplomacy, and Theatre. *MedEdPORTAL* 2018;14:10672.

For peer review only

23. Forbat L, Simons J, Sayer C, et al. Training paediatric healthcare staff in recognising, understanding and managing conflict with patients and families: findings from a survey on immediate and 6-month impact. *Arch Dis Child* 2017;102(3):250-4.

24. Ministry of Healthcare and Welfare T. Care Provided by Mediator in Medical Dispute: An Official Course Handbook. Taipei, Taiwan: Ministry of Healthcare and Welfare; 2021. Available from: https://medcare.tdrf.org.tw/educational-learning/#edu5.

25. Saundry R, Mcardle L, Thomas P. Reframing workplace relations? Conflict resolution and mediation in a primary care trust. *Work Employ Soc* 2013;27(2):213-31.

26. Harmon KMJ. The Effective Mediator. *J PROF ISS ENG ED PR* 2006;132(4):326-33.

27. Waldman EA. Identifying the Role of Social Norms in Mediation: A Multiple Model Approach. *Hastings Law J* 1997;48(4):703.

28. Richards T. Chasms in communication. *Brit Med J* 1990;301:1407–8.

29. Studdert DM, Mello MM, Sage WM, et al. Defensive medicine among high-risk specialist physicians in a volatile malpractice environment. *Jama* 2005;293(21):2609-17.

30. Al Shamsi H, Almutairi AG, Al Mashrafi S, et al. Implications of Language Barriers for Healthcare: A Systematic Review. *Oman Med J* 2020;35(2):e122.

31. Studdert DM, Brennan TA. No-fault compensation for medical injuries: the prospect for error prevention. *Jama* 2001;286(2):217-23.

32. Jones S. Putting the Person into Person-Centered and Immediate Emotional Support. *Commun Res* 2016;31(3):338-60.

33. Ha JF, Longnecker N. Doctor-Patient Communication: A Review. *Ochsner J* 2010;10:38–43.

34. De Gelder B, De Borst AW, Watson R. The perception of emotion in body expressions. *Wiley Interdiscip Rev Cogn Sci* 2015;6(2):149-58.

For peer review only

35. Benbassat J, Baumal R. What is empathy, and how can it be promoted during clinical clerkships? *Acad Med* 2004;79(9):832-9.

36. Platt FW. Empathy: can it be taught? *Ann Intern Med* 1992;117(8):700; author reply 1.

37. Hardee JT. An Overview of Empathy: Perm J. 2003 Fall;7(4):51-4.

38. Stroebe M, Schut H, Boerner K. Cautioning Health-Care Professionals. *Omega (Westport)* 2017;74(4):455-73.

39. Brunjes GB. Spiritual pain and suffering. *Asian Pac J Cancer Prev* 2011;11(Suppl 1):31-6.

40. Ohrnberger J, Fichera E, Sutton M. The relationship between physical and mental health: A mediation analysis. *Soc Sci Med* 2017;195:42-9.

41. Mcknight PE, Monfort SS, Kashdan TB, et al. Anxiety symptoms and functional impairment: A systematic review of the correlation between the two measures. *Clin Psychol Rev* 2016;45:115-30.

42. Mcknight PE, Kashdan TB. The importance of functional impairment to mental health outcomes: a case for reassessing our goals in depression treatment research. *Clin Psychol Rev* 2009;29(3):243-59.

43. Wilkins CH. Effective Engagement Requires Trust and Being Trustworthy. *Med Care* 2018;56 Suppl 10 Suppl 1(10 Suppl 1):S6-s8.

44. Wu Y, Jiang F, Ma J, et al. Experience of Medical Disputes, Medical Disturbances, Verbal and Physical Violence, and Burnout Among Physicians in China. *Frontiers in Psychology* 2021;11.

45. Hogg R, Hanley J, Smith P. Learning lessons from the analysis of patient complaints relating to staff attitudes, behaviour and communication, using the concept of emotional labour. *J Clin Nurs* 2018;27(5-6):e1004-e12.

For peer review only

46. Bush RAB, Folger JP. The Promise of Mediation: Responding to Conflict Through Empowerment and Recognition: San Francisco, CA: Jossey-Bass; 1994.

47. Sally H. Transformative Mediation and Gestalt: The Good Form of Empowerment and Recognition: A Response to Lisa Gaynier. *Gestalt rev* 2003;7(3):204-9.

48. Epstein RM, Franks P, Fiscella K, et al. Measuring patient-centered communication in patient-physician consultations: theoretical and practical issues. *Soc Sci Med* 2005;61(7):1516-28.

49. Kumar S. Burnout and Doctors: Prevalence, Prevention and Intervention. *Healthcare (Basel)* 2016;4(3).

50. Lemaire JB, Wallace JE. Burnout among doctors. *Bmj* 2017;358:j3360.

51. Albert W W. Medical error: the second victim. The doctor who makes the mistake needs help too. *Brit Med J* 2000;320(7237):726-7.

52. Mitzman J, Jones C, Mcnamara S, et al. Curated Collection for Educators: Five Key Papers About Second Victim Syndrome. *Cureus* 2019;11(3):e4186.

53. Tamburri LM. Creating Healthy Work Environments for Second Victims of Adverse Events. *AACN Advanced Critical Care* 2017;28(4):366-74.

54. Ullstrom S, Andreen Sachs M, Hansson J, et al. Suffering in silence: a qualitative study of second victims of adverse events. *BMJ Qual Saf* 2014;23(4):325-31.

55. Kerasidou A, Horn R. Making space for empathy: supporting doctors in the emotional labour of clinical care. *BMC Med Ethics* 2016;17:8.

For peer review only - http://bmjopen.bmj.com/site/about/guidelines.xhtml

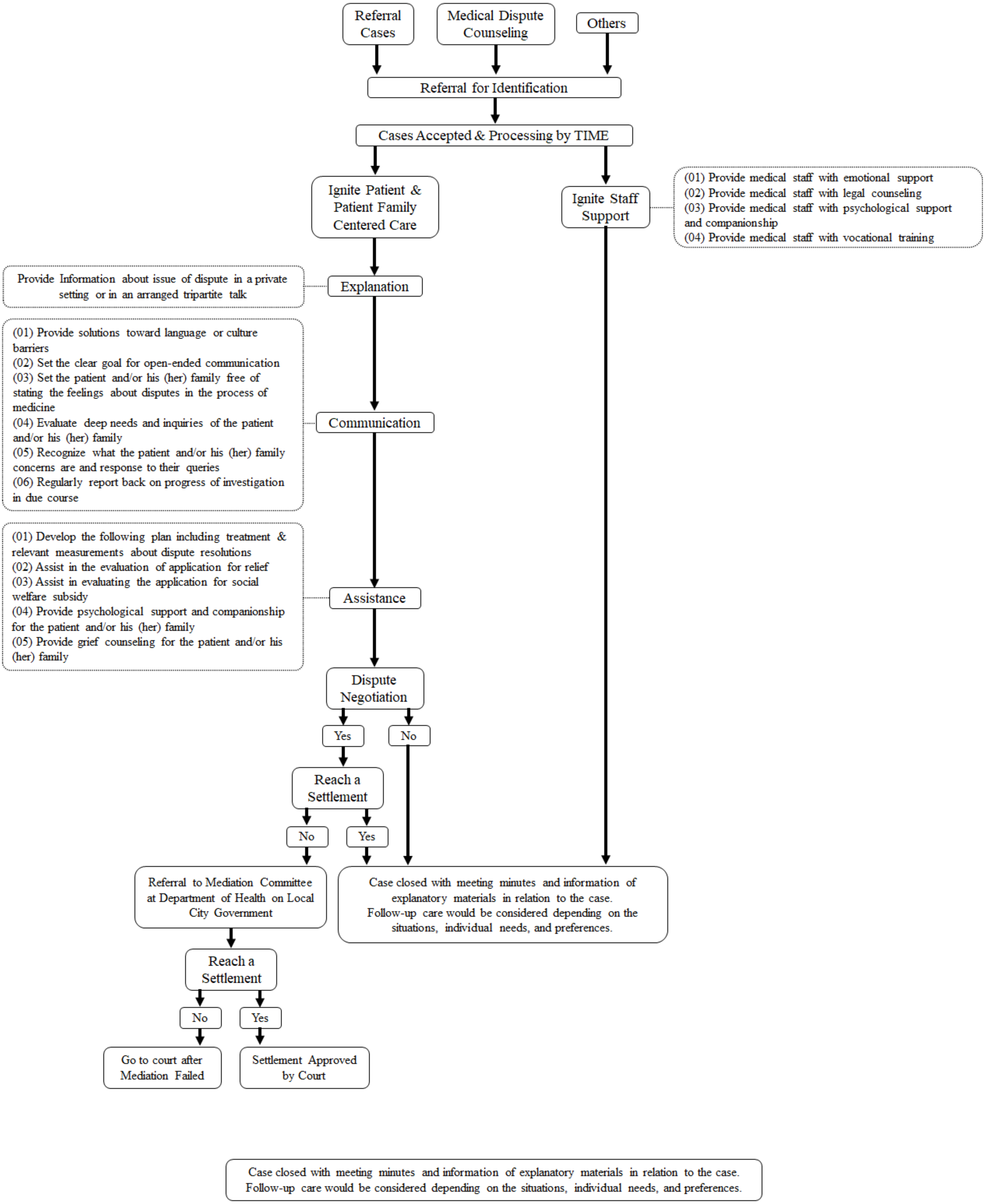

Figure 1. Flowchart of the execution of the triple-I mediation (TIME) programme.

If a dispute case is referred for dispute consultation, mediators attend the relevant location and collaborate with their team members of different specialties depending on the scale and type of the dispute. As a fair and objective third party, they provide their services equally to both patients and medical staff. If a settlement is not reached, the dispute case is referred to a mediation committee at the department of health of the local city government for further case review and processing. If both medical staff and patients cannot reach a final agreement, the case is sent for a trial for a final decision to be made by a judge.

The checklist of the Conducting and REporting of DElphi Studies (CREDES)

		Page location in the main document
Rationale for the choice of the Delphi technique	01. Justification	6
Planning and design	02. Planning and process	6-11
	03. Definition of consensus	7
Study conduct	04. Informational input	8-11
	05. Prevention of bias	7-11
	06. Interpretation and processing of results	9-11
	07. External validation	11
Reporting	08. Purpose and rationale	6-7, 9-11
	09. Expert panel	8
	10. Description of the methods	7-11
	11. Procedure	7-11
	12. Definition and attainment of consensus	9-11
	13. Results	12
	14. Discussion of limitations	17
	15. Adequacy of conclusions	17
	16. Publication and dissemination	11

For peer review only
